# Thermally Activated Delayed Fluorescence: Beyond the Single Molecule

**DOI:** 10.3389/fchem.2020.00716

**Published:** 2020-09-18

**Authors:** Marc K. Etherington

**Affiliations:** Department of Mathematics, Physics and Electrical Engineering, Northumbria University, Newcastle upon Tyne, United Kingdom

**Keywords:** thermally activated delayed fluorescence (TADF), aggregation, solid state solvation effect, photophysics, organic light-emitting diodes (OLEDs)

## Abstract

Emitters that exhibit thermally activated delayed fluorescence (TADF) are of interest for commercial applications in organic light-emitting diodes (OLEDs) due to their ability to achieve internal quantum efficiency of 100%. However, beyond the intrinsic properties of these materials it is important to understand how the molecules interact with each other and when these interactions may occur. Such interactions lead to a significant red shift in the photoluminescence and electroluminescence, making them less practicable for commercial use. Through summarizing the literature, covering solid-state solvation effects and aggregate effects in organic emitters, this mini review outlines a framework for the complete study of TADF emitters formed from the current-state-of-the-art techniques.

## Introduction

Increasing the efficiency and stability of organic light-emitting diodes (OLEDs) is a focus of significant attention for researchers and one aspect of improving these systems is to produce novel emitters that have internal quantum efficiencies (IQEs) above the 25% dictated by spin statistics (Baldo et al., 1999). Thermally activated delayed fluorescence (TADF) (Uoyama et al., 2012; Dias et al., 2013) is one phenomenon that is used to achieve 100% IQE (Lin et al., 2016; Liu et al., 2018b; Zeng et al., 2018). This significant increase arises from the molecule being able to promote non-emissive triplet states to the emissive singlet state *via* reverse intersystem crossing (rISC). The rISC process requires a small energy gap between the singlet and triplet state and this can be achieved in a variety of ways and through a variety of molecular designs: exciplex systems where the donor (D) and acceptor (A) are non-identical molecules;(Sarma and Wong, 2018; Colella et al., 2019; Tang et al., 2020) organometallic systems (Di et al., 2017; Conaghan et al., 2018; Yersin et al., 2018; Mahoro et al., 2020) metal halide perovskites;(Zhou and Yan, 2019; Qin et al., 2020) and fully organic systems where the D and A are covalently bonded. (Uoyama et al., 2012; Dias et al., 2013) The subjects of this review are fully organic covalently bonded donor–acceptor (D–A) systems or donor–acceptor–donor (D–A–D) systems. D–A and D–A–D systems have the highest occupied molecular orbital (HOMO) and lowest unoccupied molecular orbital (LUMO) localized on the D and A units respectively to produce charge-transfer (CT) states and the required small singlet–triplet gap. This localization is achieved through control of the dihedral angle between the D and A moieties and the relative electron donating and accepting strengths of the D and A units.

TADF systems typically emit from singlet charge-transfer states (^1^CT), which are almost isoenergetic with triplet charge-transfer states (^3^CT), however intersystem crossing, and rISC, between these two states is prohibited (Lim and Kedzierski, 1973). This limitation can be overcome by coupling to a third state that does not have the same character. For most TADF systems this third state is a close-lying locally excited triplet state (^3^LE). Through spin-vibronic coupling of the ^3^LE state with the ^3^CT state the rISC process between ^3^CT and ^1^CT is enhanced. This was demonstrated theoretically by Gibson et al. (2016) and experimentally by Etherington et al. (2016). The need for proximity of the ^3^LE state with the ^3^CT state provides a new criterion for the molecular design of TADF systems. One major impact of this result is that to design a TADF emitter with a particular emission wavelength, the choice of D and A units is now limited by their ^3^LE energy.

Most work on TADF systems, through photophysical studies and within devices, is performed either in the solution state or dilute concentrations within small molecule or polymer hosts, meaning that the measurements are in an ideal scenario and relate predominantly to a single, isolated molecule. However, the CT states are also susceptible to solvent polarity or solid-state host rigidity, which can influence the emissive and functional behavior of the compound and there are many research articles and reviews on this topic (Etherington et al., 2016; Santos et al., 2016a,b, 2018; Haseyama et al., 2017; Wong and Zysman-Colman, 2017; Chatterjee and Wong, 2019; Hung et al., 2019). Although it must be noted that there is an extensive field of research into exciplex systems and controlling fluorescence behavior through host–guest interactions, where intermolecular interactions are embraced. This literature considers the interactions between non-identical molecules to achieve the small exchange energies and high rISC rates required for TADF (Kim et al., 2016; Nakanotani et al., 2016; Sarma and Wong, 2018; Chatterjee and Wong, 2019; Tang et al., 2020) and it has been observed that host–guest interactions can also imbue functional properties (Matsunaga and Yang, 2015; Feng et al., 2017; Ono et al., 2018).

Equally important is a molecule's interaction with identical molecules. There is extensive literature concerning aggregation-induced emission (AIE) of organic systems (Luo et al., 2001; Hong et al., 2009; Mei et al., 2014) and these effects need to be considered for TADF systems. This review will give an overview of the phenomena that occur when we consider TADF emitters beyond the ideal of the single molecule as well as the techniques used to study them. This review will focus on novel compounds that are designed or used for their behavior in aggregates and how studying the molecules' mechanochromism, thermochromism and concentration effects in doped films facilitate the understanding of new functional behaviors. The review will also reflect on the discovery of functional behavior in existing compounds, especially 1,2,3,5-tetrakis(carbazol-9-yl)-4,6-dicyanobenzene (**4CzIPN**)–an archetypal TADF emitter (Uoyama et al., 2012) and how this may influence the interpretation of previous results highlighting the two effects related to the red shifting of emission with concentration: solid-state solvation effect (SSSE) and aggregation.

## Concentration and Aggregation in Organic Emitters

Aggregation-caused quenching (Thomas et al., 2007) and AIE (Luo et al., 2001; Hong et al., 2009; Mei et al., 2014) are two phenomena that provide a framework for the study of organic emitters beyond the single molecule in neat films, organic crystals and doped matrices. The basis of the AIE field is to understand the interaction between the molecules in these systems and establish the causes of enhancements or reductions in emission, shifts in emission color, mechanochromism or thermochromism. Recent publications and reviews in the area include (Hong et al., 2009; Mei et al., 2014; Furue et al., 2016; Gan et al., 2016; Sturala et al., 2017; Zhang et al., 2017, 2019b, 2020; Liu et al., 2018a; Yu et al., 2020), which demonstrate that phenyl rings should not be considered as entirely inert spacer units in the design of organic emitters and that the aggregation effects can be controlled through photoexcitation. These results have far-reaching implications for molecular design of organic emitters as researchers now need to take care in their choice of moieties during synthesis.

### Novel TADF Emitters Exhibiting Functional Behavior

**Figure 1:**
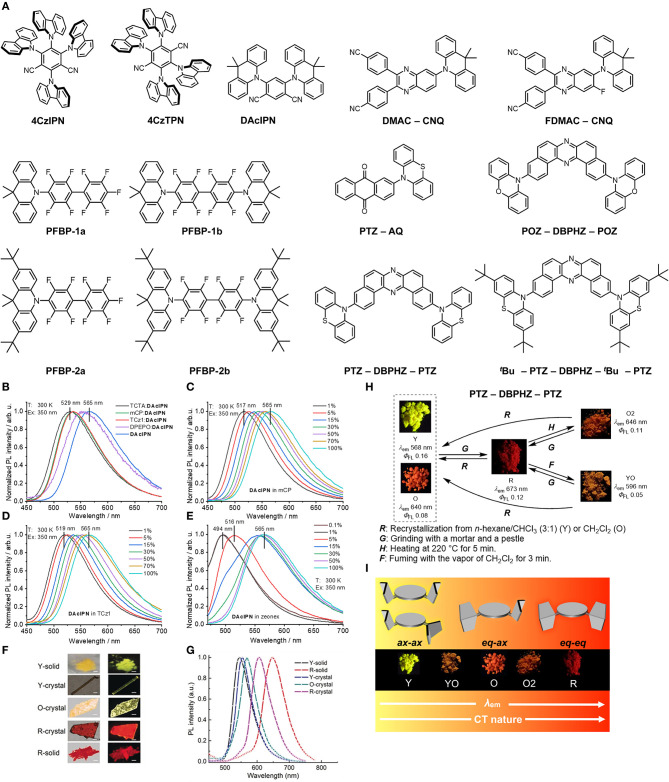
**(A)** The chemical structures of the compounds discussed in the review. **(B–E)** The effects of host and concentration on the emission profile of **DAcIPN** showing the significant red shifts observed when increasing the compound concentration. In **(B)** the shift in emission of **DAcIPN** in 15 wt% films of hosts of different polarizability showing that the red shift trend does not follow according to the polarizability of the host. **(C–E)** Red shifts in the emission occur across three different hosts, mCP, TCz1 (Tsai et al., 2007) and zeonex respectively with increasing concentration. Adapted with permission from Elsevier from Figure 4 in Skuodis et al. (2018) **(F,G)** The different emission profiles of **PTZ-AQ** as a function of solid-state environment from Y-solid to R-solid. Adapted with permission from John Wiley and Sons from Figure 1 in Huang et al. (2018) **(H)** The different forms and the methods of obtaining them for **PTZ-DBPHZ-PTZ** and **(I)** how the quasi-equatorial (eq) and quasi-axial (ax) conformers of phenothiazine influence the solid-state emission of **PTZ-DBPHZ-PTZ**. Adapted from Figures 4, 8 in Okazaki et al. (2017); (Published by The Royal Society of Chemistry).

Implementing the techniques found in the works mentioned above to TADF systems becomes somewhat more complicated as there is now not just the behavior of the prompt singlet emission to explain but also the processes and energetics underlying rISC and delayed emission. Functional phenomena in TADF systems as a result of intermolecular interactions have been observed for a range of D–A and D–A–D systems (Hladka et al., 2018; Huang et al., 2018; Klimash et al., 2018; Pashazadeh et al., 2018; Shi et al., 2018; Skuodis et al., 2018; Bhatia and Ray, 2019; Zheng et al., 2019). These works provide methodologies and insights in understanding the influence that intermolecular interactions have on TADF emitters including their photophysical properties. The compounds discussed in this review are shown in Figure 1A.

Skuodis et al. (2018) observed changes in emission behavior as a function of concentration, the host molecule, mechanical force and thermal annealing in 4,6-Di(9,9-dimethylacridan-10-yl)isophthalonitrile (**DAcIPN**). The researchers found the photoluminescence quantum yield (PLQY) of **DAcIPN** decreased from 83% at 15 wt% in 1,3-Bis(N-carbazolyl)benzene (mCP) to 47% in a film of neat **DAcIPN**–they attribute this result to a decrease in TADF contribution in the neat films. The energy of the emission red shifted with increasing concentration and was 2.32 eV (533 nm) in the mCP matrix and 2.19 eV (565 nm) in the neat film, a trend that was observed for a variety of different hosts (Figures 1B–E). The authors considered two explanations for this red shift in emission for the compound in solid-state hosts: SSSE (Bulović et al., 1999) and aggregation. They found that the red shifts did not correlate with the solid-state polarizabilities of the hosts and therefore aggregation was the dominant effect in these systems, with concomitant changes in the dihedral angle of the **DAcIPN**. During thermal annealing of the films at 130°C the authors observed a significant blue shift in the emission of both the doped and non-doped films: the doped film blue shifted from 2.19 eV (565 nm) to 2.40 eV (517 nm), which was attributed to the thermal energy allowing the molecules to disaggregate. This disaggregation allowed the dihedral angle to increase, restoring the higher energy emission. The disaggregation also caused an increase in the PLQY of the neat film from 47 to 64%.

A similar study by Hladka et al. (2018) from the same group focused on these phenomena in sky-blue emitters based on perfluorobiphenyls (PFBP). The researchers studied four systems known as **PFBP-1a**, **PFBP-1b**, **PFBP-2a**, and **PFBP-2b** and showed that **PFBP-2a**, contrary to the other compounds, showed a blue shift in emission when a thermally-evaporated film of the compound was fumed with toluene vapor. This blue shift was attributed to polymorphism of the compound and shows that very small changes in structure between a set of similar compounds can have significant effects on their aggregation properties. As polymorphism is a common explanation for many of these observed properties in the solid-state, knowledge of polymorphism and crystallography is crucial to understanding the fundamental origins of this functional behavior (Chung and Diao, 2016; Bernstein, 2020; Levesque et al., 2020). The importance of knowing the crystal structures of the compounds for understanding the packing and intermolecular interactions is demonstrated in the work by Klimash et al. (2018) In this work they uncovered differences in the molecular crystal structures and how these relate to differences in the TADF efficiency.

Polymorphism as one of the causes of different TADF behavior in crystalline films/powders is reported by Zheng et al. (2019) in 4,4′-(6-(9,9-dimethylacridin-10(9H)-yl)quinoxaline- 2,3-diyl)dibenzonitrile (**DMAC-CNQ**) and 4,4′-(6-(9,9-dime- thylacridin-10(9H)-yl)-7-fluoroquinoxaline-2,3-diyl)dibenzo- nitrile (**FDMAC-CNQ**). The authors reported two different polymorphs for **DMAC-CNQ**, which they termed Y-crystal and O-crystal and three polymorphs for **FDMAC-CNQ** termed Y-crystal, O-crystal and R-crystal based on the emission color (Yellow, Orange and Red). The changes in emission color between the crystals are due to different conformations of the DMAC moiety that then has a resultant effect on the π-π interactions in the crystal. The trend for both systems is that, going from yellow to red, the number of π-π interactions increases as a function of the DMAC conformation. Similarly, a trend of decreasing TADF contribution and PLQY was observed going from Y-crystal to R-crystal, again related to the conformations of the compound. These single crystal studies which are linked to the molecular structure has allowed the authors to interpret the mechanochromism behavior they observed from the pristine, ground and fumed states of the compound. Single crystal X-ray diffraction and powder X-ray diffraction studies provide an important tool for the study of TADF beyond the single molecule and are a key part of the framework in determining aggregation effects.

Huang et al. (2018) demonstrated the significant accumulation of functional behavior that can be obtained in TADF D–A molecules. In the compound 2-(phenothiazine-10-yl)-anthraquinone (**PTZ-AQ**) they observed TADF, aggregation-induced emission and mechanochromism alongside polymorphism. They comment on the effect that aggregation has on the TADF behavior arguing that TADF can even be tuned by the aggregation state. Figures 1F,G show the different emission profiles that can be obtained as a function of solid-state environment. The crystals and solids, which the authors categorize into five different aggregation states, facilitate emission energies from the green to the red: a yellow solid (Y-solid), a red solid (R-solid), a yellow rod-like crystal (Y-crystal), an orange flake-like crystal (O-crystal) and a red flake-like crystal (R-crystal) with the R-crystal showing the smallest singlet-triplet gap and high PLQY.

In general, the idea of molecular conformation affecting the TADF and mechanochromism properties of a compound is in agreement with the work of Okazaki et al. (2017) In 2017 the researchers compared phenoxazine–dibenzo[*a,j*]phenazine–phenoxazine (**POZ–DBPHZ–POZ**) and the phenothiazine analog with *tert*-butyl groups (^***t***^**Bu-PTZ–DBPHZ–**^***t***^**Bu-PTZ**) and (**PTZ–DBPHZ–PTZ**) without *tert*-butyl groups. Although two polymorphs of **PTZ–DBPHZ–PTZ** were found only one of ^***t***^**Bu-PTZ–DBPHZ–**^***t***^**Bu-PTZ** was discovered. In this study the emission of both compounds red shifted with grinding. Upon heating the emission of ^***t***^**Bu-PTZ–DBPHZ–**^***t***^**Bu-PTZ** continued to red shift however, the emission of **PTZ–DBPHZ–PTZ** blue shifted (Figure 1H). The particular mechanochromic behavior of **PTZ–DBPHZ–PTZ** is linked to the tendency for PTZ to form as one of two conformers (Figure 1I): quasi-axial (ax) or quasi-equatorial (eq). (Malrieu and Pullman, 1964; Bodea and Silberg, 1968; Coubeils and Pullman, 1972; Etherington et al., 2017; Santos et al., 2018) The red emission comes from the highly twisted equatorial-equatorial conformer, the orange from the axial-equatorial and the yellow emission from the axial-axial conformer which allows formation of a higher energy ^1^LE state. This again demonstrates the importance of linking mechanochromic and TADF behaviors to the molecular structure through single crystal X-ray diffraction techniques. Mechanochromism is a useful tool and part of the framework for studying intermolecular effects in TADF systems. The work by Pashazadeh et al. (2018) shows how TADF can be turned on and off with grinding and how the intermolecular interactions helped mediate the singlet triplet gaps and control the fundamental emission phenomena.

### Uncovering Intermolecular Effects in Existing Motifs

As noted in the work by Skuodis et al. (2018) there are two proposed origins for the red shift with increasing concentration in solid-state hosts: the SSSE effect (Cotts et al., 2017; Delor et al., 2017; Han and Kim, 2019) and aggregation (dimer or intermolecular) effects (Skaisgiris et al., 2019; Zhang et al., 2019a) In this section of the review, literature surrounding an existing and widely-used motif, **4CzIPN**, (Uoyama et al., 2012) will be used to contextualize this red shift in emission.

**Figure 2:**
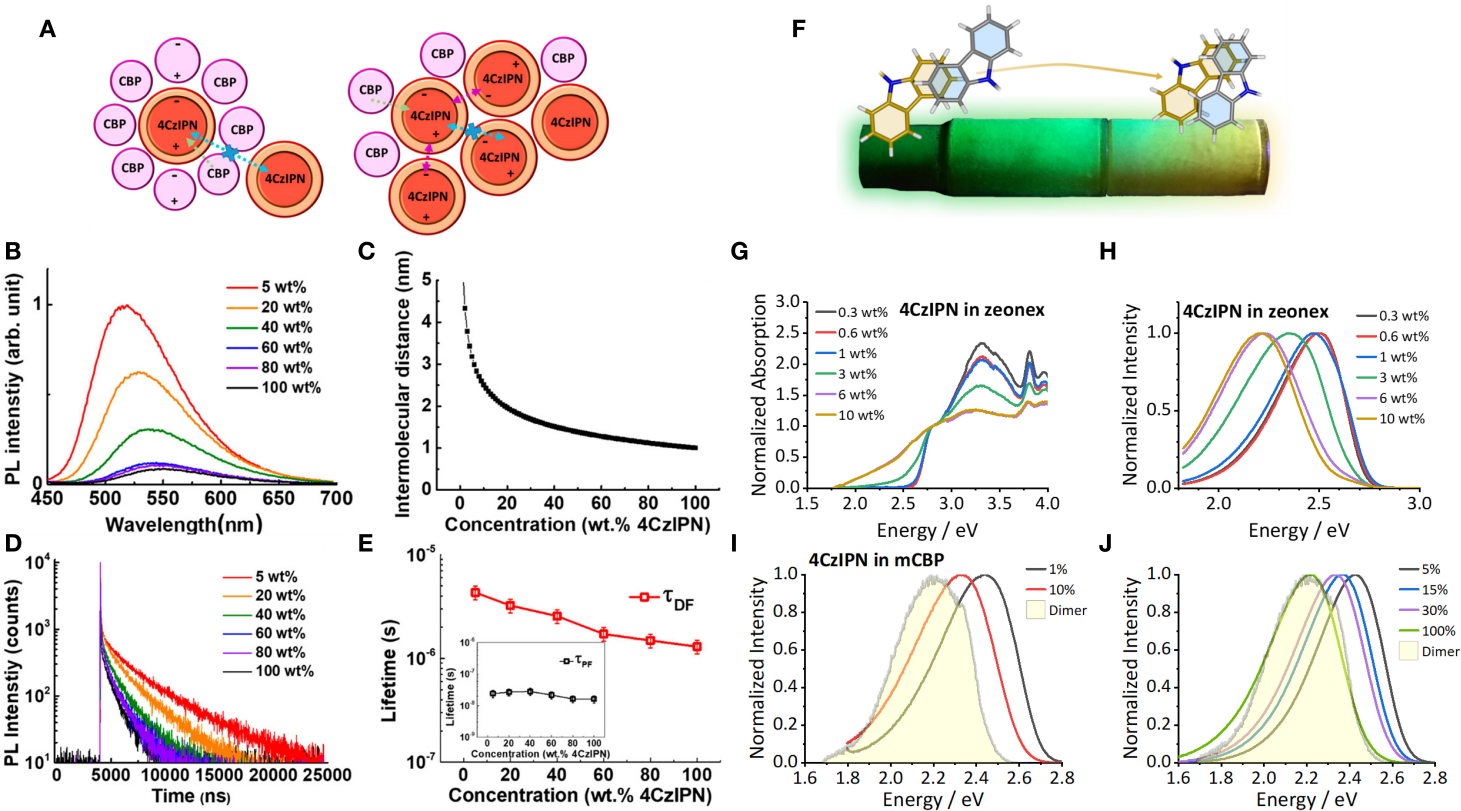
**(A)** A diagram showing the effect of increasing concentration on the processes of **4CzIPN**. The dipole moment of **4CzIPN** is larger than CBP and affects the emission color of neighboring **4CzIPN** molecules through SSSE. **(B–E)** Photophysical studies of **4CzIPN** in evaporated films of CBP with increasing concentration. **(B)** There is a reduction in intensity and a bathochromic shift in the emission color and **(C)** a reduction in the intermolecular distance that leads to **(D,E)** a reduction in the delayed fluorescence lifetime (τ_DF_). Figures adapted with permission from Kim et al. (2017). “Concentration Quenching Behavior of Thermally Activated Delayed Fluorescence in a Solid Film.” The Journal of Physical Chemistry C 121 (26): 13986–13997. https://doi.org/10.1021/acs.jpcc.7b02369. Copyright 2017 American Chemical Society (Kim et al., 2017) **(F)**. The effects of dimers on the emission color of **4CzIPN** that was demonstrated under UV illumination in a sublimation tube. **(G)** The appearance of the absorption band of the dimer species with increasing concentration in a zeonex film and **(H)** the resultant effect on photoluminescence. The presence of the new absorption band allows direct excitation of the dimer species, which has characteristic emission (shaded area) that appears in **(I)** the photoluminescence of evaporated films of 3,3′-Di(9H-carbazol-9-yl)-1,1′-biphenyl (mCBP) and **(J)** the electroluminescence of devices using a proprietary material from Merck as the host. Figures adapted from Etherington et al. (2019).

In 2017 Kim et al. (2017) studied the effects of concentration on the emission profile and TADF characteristics of **4CzIPN**. They observed a significant redshift in the emission with increasing concentration (Figures 2A–E) and a decrease in the TADF lifetime. These red shifts, both in photoluminescence and electroluminescence, have been observed by a range of researchers (Nakanotani et al., 2013; Kim and Lee, 2014; Komatsu et al., 2015; Wang et al., 2016; Zhu et al., 2017; Li et al., 2018; Niwa et al., 2018). Kim et al. (2017) attributed these red shifts and the consequential change in TADF behavior to the changing dipole moment of the surrounding matrix as the host molecules are subsequently replaced with more **4CzIPN** molecules (Figure 2A). **4CzIPN** is more polar than the host 4,4′-Bis(N-carbazolyl)-1,1′-biphenyl (CBP) and therefore as the concentration increases, the dipole moment and polarizability of the environment increases producing a red shift in the emission. This is the effect known as SSSE (Bulović et al., 1999).

Performing measurements in solid-state hosts with different polarities like Skuodis et al. (2018) is one way of deconvoluting SSSE and aggregation effects, however to completely characterize these systems a diverse framework of measurements is required. In 2019, Etherington et al. (2019) performed a study on **4CzIPN**, incorporating techniques used in the majority of investigations mentioned in section Novel TADF Emitters Exhibiting Functional Behavior, including mechanochromism, thermochromism, single-crystal X-ray diffraction and combined them with time-resolved photoluminescence spectroscopy. This framework uncovered the dimer species of **4CzIPN** present in certain environments. The effects of these intermolecular interactions were seen during sublimation of **4CzIPN** and Etherington et al. observed that crystals and solid-state powders with different emission colors were produced along the sublimation tubing (See Figure 2F). This emission color was found to be changeable through thermal annealing and the introduction of mechanical energy. The fact that in neat films there were changes in emission color without a change in polarizability of the surrounding media suggests that SSSE is not the sole, or even dominant, effect in determining the emission properties of **4CzIPN**. Indeed, the related material 2,3,5,6-Tetra(carbazol-9-yl)benzene-1,4-dicarbonitrile (**4CzTPN)** (Uoyama et al., 2012) is known to give emission of orange-red within the solid-state but only yellow emission in one of the most polar solvents dimethyl sulfoxide (DMSO). This result strongly suggests that polarizability is not the most crucial effect for the emission properties of these materials in the solid-state. Theoretical work by Northey et al. (2017) shows that the SSSE, while present in TADF systems, can be restricted due to rigidity of the host molecule in a solid-state environment.

The dimer/aggregate species observed in **4CzIPN** through excitation at 2.33 eV (532 nm), significantly below the band gap of the monomer absorption band (Figure 2G), has an emission profile that almost exactly matches the higher concentration zeonex films and the neat film (Figures 2H–J). This gives unequivocal evidence that intermolecular interactions play a key role in determining the emission properties, color purity and TADF efficiency of these systems. The framework Etherington et al. established (Etherington et al., 2019), which builds upon techniques in the wider literature (section Novel TADF Emitters Exhibiting Functional Behavior) provides the means to deconvolute SSSE from intermolecular effects. This is an important aspect to build the complete picture of TADF behavior beyond the single molecule while highlighting the need for researchers to be acutely aware of intermolecular interactions in future studies.

## Conclusion and Outlook

While there has been vast development of new TADF emitters and our understanding of the fundamental processes of rISC and the spin states has developed, it is now time to look towards understanding these processes in non-ideal situations using the techniques and framework discussed above. This framework will facilitate the development and understanding of functional materials that are sensitive to mechanical and thermal energy while opening up pathways to AIE-based, non-doped OLEDs (Furue et al., 2016; Liu et al., 2018a; Cai et al., 2020; Yang et al., 2020). It will also help unify the study of intramolecular TADF emitters with the studies of polymorphism (Chung and Diao, 2016; Bernstein, 2020; Levesque et al., 2020) exciplexes (Kim et al., 2016; Sarma and Wong, 2018; Tang et al., 2020) and host–guest interactions (Matsunaga and Yang, 2015; Feng et al., 2017; Ono et al., 2018).

The framework should continually incorporate new experimental techniques that are sensitive to the environmental changes underpinning this behavior to be controlled. So far, the framework includes single crystal studies and controlled mechanochromic and thermochromic analyses. This framework will then allow for the control and use of these properties as demonstrated in the work by Bhatia and Ray (2019) The researchers utilized the dimer/aggregate state, not to improve TADF but rather, to introduce room temperature phosphorescence. A combination of the emission profiles of the monomer, dimer and aggregated species produces a wide band white afterglow.

Future work will specifically link molecular structure to the desired properties. Sussardi et al. have begun this progress linking the crystal structure of a compound directly to the emission profile as a function of pressure (Sussardi et al., 2020). This work allows a systematic way to study and correlate the intermolecular interactions and the photophysics. Etherington et al. (2019) showed that the addition of tert-butyl groups do little to prevent the intermolecular interactions and thus we must look to bulkier spacer units or alternative methods to control these effects. High-pressure crystallography, similar to the work of Sussardi et al. (2020) would be able to test the limit of these bulky units. The techniques and studies mentioned in this review provide a basis to develop future frameworks and criteria for inhibiting or enhancing intermolecular interactions. This will help to provide high color purity compounds for OLED commercialization and unlock new functional behavior for applications beyond OLEDs.

## Author Contributions

MKE devised the idea for the topic of this review, collated the literature and wrote the review. The author confirms being the sole contributor of this work and has approved it for publication.

## Conflict of Interest

The author declares that the research was conducted in the absence of any commercial or financial relationships that could be construed as a potential conflict of interest.
